# Towards patient‐centred communication in the management of older patients' medications across transitions of care: A focused ethnographic study

**DOI:** 10.1111/jocn.16162

**Published:** 2021-12-06

**Authors:** Guncag Ozavci, Tracey Bucknall, Robyn Woodward‐Kron, Carmel Hughes, Christine Jorm, Elizabeth Manias

**Affiliations:** ^1^ Centre for Quality and Patient Safety Research The School of Nursing and Midwifery Institute for Health Transformation Alfred Health Deakin University Burwood Vic. Australia; ^2^ Department of Medical Education Melbourne Medical School The University of Melbourne Parkville Vic. Australia; ^3^ School of Pharmacy Queen's University Belfast Belfast UK; ^4^ School of Public Health The University of Sydney Camperdown NSW Australia

**Keywords:** aged, decision‐making, focused ethnography, nursing, health communication, medication therapy management, older patients, patient participation, patient preference, patient transfer

## Abstract

**Background:**

Communication about managing medications during transitions of care can be a challenging process for older patients since they often have complex medication regimens. Previous studies highlighted that links between communication breakdowns and medication incidents in older patients occur mainly at discharge or in the post‐discharge period. Little attention has been paid to exploring communication strategies facilitating patient‐centred medication communication at transitions of care from a discourse‐analytic perspective.

**Objectives:**

To explore, through a discursive lens, strategies that enable patient‐centred medication communication at transitions of care.

**Design:**

A focused ethnographic study was employed for this study. The study was reported according to the COREQ checklist.

**Methods:**

Interviews, observations and focus groups were analysed utilising Critical Discourse Analysis and the Medication Communication Model following thematic analysis. Data collection was undertaken in eight wards across two metropolitan hospitals in Australia.

**Results:**

Patient preferences and beliefs about medications were identified as important characteristics of patient‐centred communication. Strategies included empathetic talk prioritising patients' medication needs and preferences for medications; informative talk clarifying patients' concerns; and encouraging talk for enhancing shared decision‐making with older patients. Challenges relating to the use of these strategies included patients' hearing, speech or cognitive impairments, language barriers and absence of interpreters or family members during care transitions.

**Relevance to clinical practice:**

To enhance medication communication, nurses, doctors and pharmacists should incorporate older patients' preferences, previous experiences and beliefs, and consider the challenges faced by patients across transitions. Strategies encouraging patients' contribution to decision‐making processes are crucial to patient‐centeredness in medication communication. Nurses need to engage in informative talk more frequently when administering the medications to ensure older patients' understanding of medications prescribed or altered in hospital settings.

## 1 INTRODUCTION

Transitions of care involve older patients' movements between different settings and they also comprise consultations with various health professionals (World Health Organization, 2019). Communication breakdowns arising from movements across transitions of care are responsible for approximately 50–60% of hospital‐related medication incidents including incomplete or inaccurate medication records, inappropriate or delayed medication administration, and about 20% of adverse drug events, which are events that lead to patient harm (American Pharmacists Association & American Society of Health‐System Pharmacists, 2012). Older patients experience communication problems with their medications across transitions of care because of multiple interactions with health professionals, difficulties in retaining information about medication changes, and perceived lack of support (Ellins et al., 2012; Jeffs et al., 2017).

Patient‐centred communication, where health professionals engage with patients and their families and provide medication information that they can understand, can help to facilitate patient safety (World Health Organization, 2019). This process comprises health professionals responding to patients' informational needs, preferences and concerns, being sensitive to patients' emotions and beliefs, providing empathy and support, and enabling patients' self‐management and involvement in decision‐making (De Haes & Bensing, 2009; Epstein & Street, 2007; Ganz et al., 2014; Hashim, 2017). Barriers to patient‐centred communication with older patients include cognitive barriers (Grealish et al., 2019), language barriers (Nkrumah & Abekah‐Nkrumah, 2019), memory deficits (Anna & Simon, 2018), and ageist or task‐oriented communication styles (Ayalon & Tesch‐Römer, 2018; Belcher et al., 2006).

Previous studies examining patient‐centred communication have included interviews with patients and observations of clinical practice where older patients participated across transitions of care (Feder et al., 2019; Lenaghan, 2019). Studies showed that older patients were an important source of information about medication changes across settings; however, they often had limited opportunities to discuss their views or preferred to keep quiet to avoid potential disagreements with health professionals (Baillie et al., 2014; Rustad et al., 2016). Older patients' participation in decisions needed ongoing and individualised support from health professionals including recognition of patients' capacity and participation preferences (Dyrstad et al., 2015; Sawan et al., 2020). This support included tailoring information and involving families in admission and discharge communication (Dyrstad et al., 2015).

There has been a lack of in‐depth exploration of communication strategies facilitating patient‐centred medication communication from a discourse‐analytic perspective. This perspective can provide a critical lens to understand how communication occurs in various social and cultural contexts. There has also been limited research investigating different health professionals' approaches to patient‐centred medication communication (Ozavci et al., 2020). Past studies have involved focusing on a single transition point such as discharge (Allen et al., 2018; Rognan et al., 2021; Tobiano et al., 2021), or on interactions with a single health professional group such as patient‐pharmacist interactions (Braaf et al., 2015) or patient‐doctor interactions (Kripalani et al., 2007). Additionally, little attention has been placed on exploring discourses used by health professionals and older patients during medication interactions.

The aim of this study was to explore, through a discursive lens, strategies that enable patient‐centred medication communication at transitions of care.

## 2 METHODS

### 2.1 Research design

A focused ethnographic study was undertaken, with semi‐structured interviews, observations and reflexive focus groups. Focused ethnography has evolved as a pragmatic form of conventional ethnography, which enables researchers to investigate a specific social phenomenon in a particular context for an identifiable period of time, and with a pre‐established goal in mind (Knoblauch, 2005; Mayan, 2009; Roper & Shapira, 2000). Unlike conventional ethnography, the researcher who uses a focused ethnographic approach examines a distinct problem and concentrates on particular research questions that are formulated before going into the field (Christine et al., 2009; Higginbottom et al., 2013). In focused ethnography, the researcher is familiar with the research context and can conduct short‐term field visits supported by audio‐visual technologies to collect large amounts of data (Higginbottom et al., 2013; Simonds et al., 2012). The COnsolidated criteria for REporting Qualitative studies (COREQ) checklist for qualitative research has been followed in reporting this study, see Appendix S1 (Tong et al., 2007).

### 2.2 Study setting

This study was conducted in two metropolitan hospitals comprising an acute tertiary hospital (Site 1) and a geriatric rehabilitation facility (Site 2), in Melbourne, Australia. Data were collected in two specialty medical wards and one general medical ward at the first site and two rehabilitation wards and three aged care wards at the second site.

### 2.3 Participants and sampling procedure

Patients aged 65 years or older admitted to the study wards were eligible for inclusion; those with severe dementia were excluded. Purposive sampling was undertaken to ensure adequate representation of older patients from different ages including youngest‐old (65–74), middle‐old (74–84), and oldest‐old (over 85 years). Older patients from non‐English speaking backgrounds were included in observations if they were able to verbally consent in the presence of family members or interpreters who helped them with translation. Almost 21% of older patients who participated in the observations were of non‐English speaking backgrounds. The health professionals included were nurses, pharmacists, and qualified doctors who worked in study wards at least 1 day per week. Casually employed nurses were excluded. Family members of patients who were involved in the care of the older patients, and who spoke and understood English were considered for inclusion such as older patients' children, siblings, spouses or friends. After completion of interviews and observations, focus groups were conducted with different older patients and family members to those involved in interviews and observations.

Two field researchers undertook the data collection. Patient lists were reviewed by researchers for suitable patients to be approached. These lists included information about patients' ages, gender, the admission date and reasons for hospital admission. Researchers liaised with bedside nurses and nurse unit managers to determine older patients' eligibility. Older patients were recruited after researchers provided verbal and written information about the study. Sampling of health professionals occurred purposively, based on their professional disciplines, and the length and level of professional expertise in different settings. Approximately more than half of participants who were approached consented to take part in the study.

### 2.4 Data collection

Data collection consisted of semi‐structured, face‐to‐face interviews and participant observations at both hospital sites from April 2018 to October 2019. While semi‐structured interviews were conducted with all participant groups, researchers were particularly interested in older patients' perspectives of communicating about medications across transitions of care (Table 1). An approximate length of an interview was 20 min.

**TABLE 1:** Interview, observation and reflexive focus group schedules

**Semi‐structured interview schedule**
Involvement in decisions about changes to medications before and after older patient movements between settings Preferences for what to discuss about medications with health professionals on admission, discharge, transfers within and between hospitals Experiences of how older patients talked about medications to health professionals when they moved between settings
**Observation schedule**
Presence or absence of health professionals, older patients and family members during communication encounter Different ways in which individuals interact, including use of non‐verbal and verbal cues Strategies to involve older patients in medication communication Challenges and opportunities in communicating about medications Effects of the environmental and organisational context on communicating about medications as preparations are made for older patients to move between settings. Particular effects included interruptions and distractions, health professionals' multitasking, use of computer systems during communication encounters or the ability to access interpreter services
**Reflexive focus group schedule**
Issues impacting medication communication and retention of medication information Challenges affecting communicating medication changes Involvement in medication decisions made in relation to patient movements between hospital settings

Observations were conducted during key medication‐related activities, such as medication administration, ward rounds, handovers, admission and discharge sessions (Table 1). The observational data were captured using audio recordings of researchers' observation and field notes. The researchers also took photos and documented older patients' medications from the patient medical records. After completion of interviews and observations, focus groups were undertaken at both hospital sites with different older patients and family members to those who participated in the initial interviews and observations. Focus groups were conducted to obtain patients' and family members' reflections on the key issues identified during interviews and observations. The research team determined the key issues following preliminary thematic analysis of interviews and observational data and formulated focus group schedules based on these issues. Focus groups were conducted with different patients and family members to the participants who were recruited for interviews or observations. Audio recordings were collected on all data and transcribed verbatim, and summary field notes were made after each observation.

The two researchers who collected data maintained detailed field notes and began coding during data collection. Data were collected in one ward setting at a time in order to comprehensively explore the specific contextual characteristics of a specific ward setting. Field notes and codes were regularly examined during data collection. Through conversations with the whole team and reflections on field notes and codes, data collection ceased within a particular ward setting if repeated patterns of practices, experiences and perceptions were found. This occurrence of repeated patterns of practices, experiences and perceptions was viewed as the point where data saturation had been reached. The two researchers then moved to the next ward setting for data collection. Two field researchers, the study's first author and a research fellow, from pharmacy and nursing backgrounds collected data respectively. Both researchers were female and received training in qualitative research methodology at PhD and Masters level. The researchers introduced themselves as scholars to older patients, with an interest in hearing their experiences of communicating about managing medications across transition points of care. The researchers did not have a prior relationship with any participants in this study.

### 2.5 Data analysis

Thematic analysis of transcriptions was initially undertaken (Braun & Clarke, 2006). It was an iterative and content‐driven approach, which required reading and re‐reading transcripts, listening to audio files and exploring key ideas and themes by the two field researchers. Initially, verbatim transcriptions of interviews, focus groups and observations were imported into NVivo 12 (QSR Melbourne) for data management. Then, two researchers coded each transcripts independently by reading through all transcripts line‐by‐line. Thematic analysis helped the research team to make sense the data by exploring the initial codes. The research team met fortnightly to discuss findings and identify commonalities across data. These commonalities were collated by the research team into potential themes. Once potential themes were confirmed, the researchers worked through the themes to identify the different analytical elements relating to the three dimensions of Critical Discourse Analysis (CDA) and the Medication Communication Model (Fairclough, 1992; Liu et al., 2011; Manias, 2010).

The three dimensions of CDA comprising the discursive practice level, text level, and social practice level were mapped against three levels of the Medication Communication Model (Manias, 2010), which consisted of antecedents, attributes and consequences respectively. At the discursive practice level, we investigated how the text was produced, communicated, and consumed by individuals. At the text level, we identified the content and structure of the text, and explored language devices, which helped to understand how health professionals including nurses, pharmacists and doctors communicated in response to older patients' preferences, needs and beliefs. At the social practice level, we identified implications of social relations on discursive practices (Table 2). Thematic analysis and critical discourse analysis were congruent with each other, and their combined use facilitated a complimentary approach to data analysis. Initially, thematic analysis enabled us to inductively identify patterns in the data, and to make sense of the commonalities and differences obtained in episodes of social interactions between individuals. Subsequently, critical discourse analysis was viewed as a means of examining the themes in diverse ways to determine influences affecting communication, the content and nature of communication, and sociocultural and health consequences, such as patient participation in decision‐making processes and power disparities.

**TABLE 2:** Data analysis using Fairclough's Critical Discourse Analysis (CDA) and medication communication model

Levels of critical discourse analysis (Fairclough, 1992)	Relationship of levels of analysis with the medication communication model (Manias, 2010)	Guide questions
**Discursive practice level** Aim: To explore process of text production, distribution and consumption	**Antecedents dimension** Aim: To examine sociocultural and environmental influences on medication communication	What roles, characteristics and beliefs were relevant to medication communication? What psychosocial and environmental practices were used to achieve patient‐centred communication goals?
**Text level** Aim: To explore the structure and content of the text	**Attributes dimension** Aim: To examine the way communication occurs about managing medications	Who was talking or silent in medication communication? How was body language used? How were language devices such as hedging (words used that weaken the force of the statement), modality (words that express the degree of certainty, possibility, necessity or willingness), turn‐taking (patterns of speech in which speakers talk one at a time in alternating turns) used by participants?
**Social practice level** Aim: To evaluate ideological effects and hegemonic processes in which discourse participates	**Consequences dimension** Aim: To explore social and clinical consequences of medication communication	How did discursive practices influence older patients' involvement in decision‐making process? What were the social and clinical implications of discursive practices relating to patient‐centred medication communication?

The research team created a codebook and guide questions that were formulated to facilitate the data analysis process (Table 2), which were informed by previous studies focused on medication communication between health professionals and patients (Liu et al., 2012; Manias et al., 2016). The integration of the Medication Communication Model with CDA provided researchers with a focused approach to investigating complexities around medication communication by evaluating the influences of different sociocultural and environmental dynamics. To ensure the reflexivity and trustworthiness, findings were presented to other research team members from medical, nursing, pharmacy, health communication and sociology backgrounds during the regular team meetings where all members discussed and resolved discrepancies.

### 2.6 Ethics approval

This study was approved by the health service ethics committee of the two hospital sites, HREC 212/17 and the university committee, DUHREC 2018‐067. Researchers obtained informed written consent from older patients, family members and health professionals. To maintain the confidentiality, pseudonyms were used in data excerpts.

## 3 RESULTS

**FIGURE 1:**
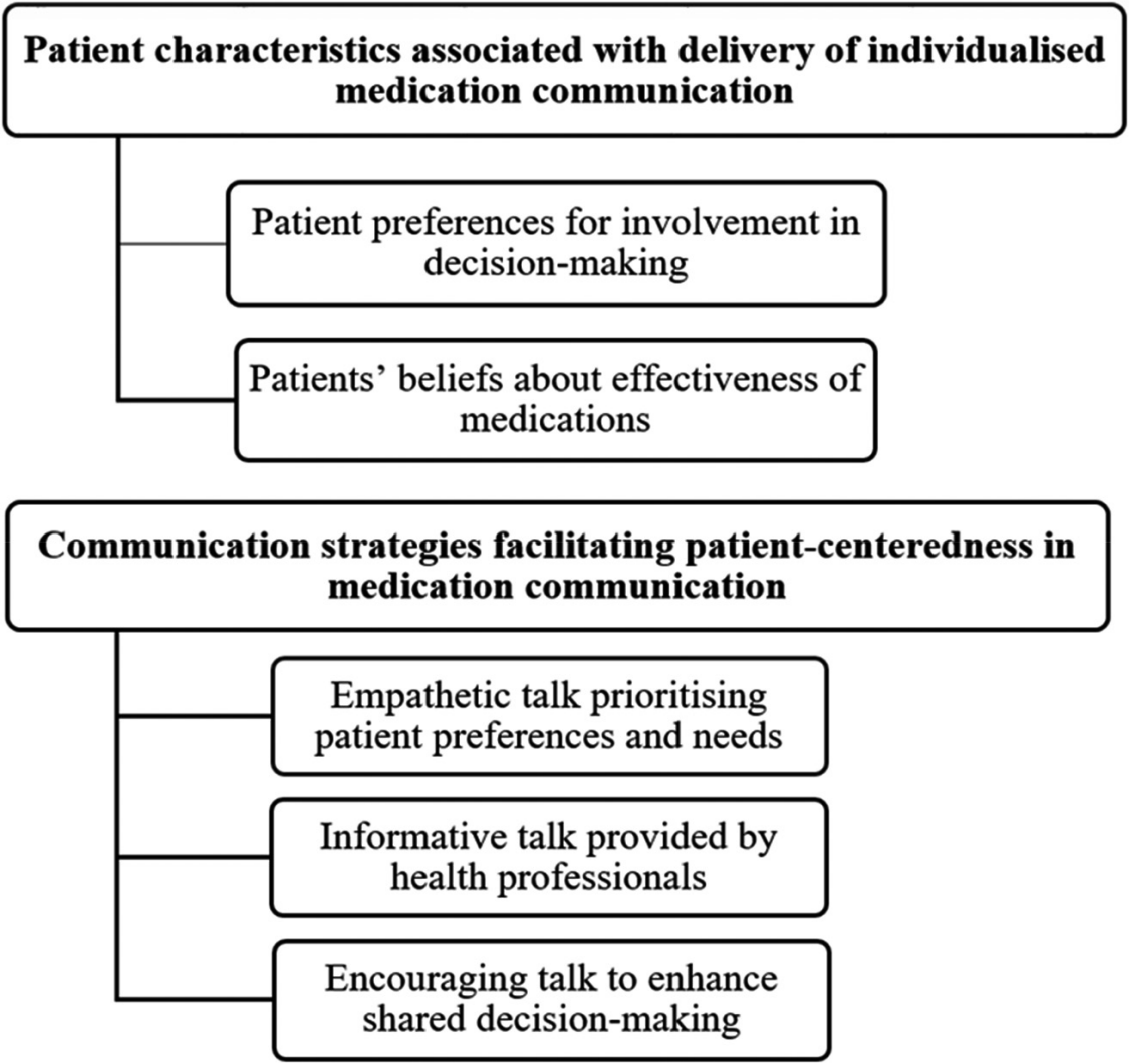
Key findings

Interviews were undertaken with 50 older patients and 29 health professionals including doctors, pharmacists, and nurses. In total, 203 h of observations were conducted with 29 health professionals and 111 older patients. Focus groups were conducted with 20 patients and 13 family members. Results were analysed from patient interviews, observations of the interactions occurred between health professionals, older patients and family members if they were present as well as focus groups with older patients and family members. Figure 1 shows the key findings.

### 3.1 Patient characteristics affecting individualised medication communication

#### 3.1.1 Patient preferences for involvement in decision‐making

Patient preferences referred to being involved in decision‐making and having a sense of control about their medications. Some older patients preferred not to participate in decision‐making after hospital admission because they trusted the expertise and knowledge of health professionals. These older patients felt adequately supported by family members or home‐care services, relied on dose administration aids, and perceived that they lacked medication knowledge or felt hesitant in challenging health professionals' expertise in the hospital environment. In the following focus group excerpt, a 70‐year‐old patient, who received support from his wife in managing medications, reflected on his involvement in medication decision‐making processes after being admitted to the geriatric rehabilitation setting:

I'll tell you the honest truth. I have complete faith in them and I just go along with what they're giving me. I've got an idea from the doctors what I'm on and I just trust the nurses to give me the right thing. (Pt15_FG15_Geri_Rehab2)

The patient did not seek an active role in medication management and preferred to defer responsibility to health professionals. He was content with information given by the doctors and the way his medications were administered by nurses, which related to his trust in health professionals' expertise. Similarly, an 86‐year‐old patient who was using a Webster pack at home indicated that she did not prefer to be involved in decision‐making in hospital: ‘When you're in hospital … it's what they do, not what I do, so you just have to accept that… I'm not a doctor, so I sort of think what right have I to be critical of that’ (Pt11_WardMed2). The patient's choice to remain passive during communication was associated with her acceptance of the dominant medical discourse of authority and her reluctance to challenge health professionals' institutional authority in medication decision‐making.

Conversely, some patients sought active involvement in medication decisions when organising to leave hospital because they wanted to take control of managing their medications after discharge. These patients were intent on self‐managing their medications at home or had experienced multiple hospitalisations in the past, and therefore were meticulous about keeping records of medication changes across transitions of care. There were older patients who demonstrated active involvement in preparing for discharge. In the following excerpt, the pharmacist encouraged the patient to use a dose administration aid (Dosette box) to facilitate his medication management after discharge because the patient has to take around twenty medications every day, whereas the patient was reluctant to do so. The interaction occurred just before the patient's discharge:

**Pharmacist turns towards patient:** So I know previously you were just self‐managing your medications and you would not pack them into a Dosette box.

**Patient:** Yeah.

**Pharmacist:** Do you feel comfortable continuing to do that?

**Patient:** Yeah

**Pharmacist:** Do you prefer that?

**Patient**: That way, I feel I'm in control. I've seen so many mix ups with the staff getting the medications, forgetting to do it. I have got my rigid [pattern] two bottles. One for morning, one for night. And last thing at night, I do medications for the following day.

**Pharmacist:** Right.

**Patient:** That way, I am in control in my life. Not somebody else trying to control my life. That's the difference.

**Pharmacist:** And you feel capable and you enjoy it maybe? A little bit, hopefully?

**Patient:** Ohhh, yes. I do enjoy it. No, it's good.

*Excerpt code: Pharm5_Obs_ Geri_Rehab2_20190527_Int2_Pt54

The patient perceived that using a pharmacist‐prepared dose administration aid would compromise his autonomy in managing medications following discharge. His statement: ‘I feel I'm in control’, indicated that he felt safer not using the aid. The patient's attempt to be actively involved demonstrated the tension between the patient discourse of self‐control and the pharmacy discourse of preventing harm.

Observations revealed that older patients were admitted to acute care settings in order to undergo a specific procedure or to stabilise their acute or chronic medical conditions. Therefore, the time that they spent in these settings was usually limited compared to those patients who were admitted to geriatric rehabilitation settings to receive more specialised and long‐term treatment to manage their complex and multiple conditions before discharge home or to residential aged care. Therefore, the time that pharmacists spent with older patients in geriatric rehabilitation settings was relatively longer than with those who were admitted to acute care settings. Within geriatric rehabilitation settings, pharmacists were able to dedicate more time with these patients to discuss about their medications. Additionally, pharmacists' education in acute care settings tended to be more rushed due to the relatively high flow of patients who were just admitted or those who needed to be discharged. There was reduced time available for exploring patient preferences during communication encounters in acute settings since pharmacists were heavily engaged in a discourse of preparedness by organising medication lists on computers before visiting patients prior to discharge.

Availability of nurses and doctors to communicate with older patients about medications in both acute and geriatric rehabilitation settings was limited due to multitasking and time constraints. Older patients were observed to miss opportunities where they could express their medication preferences. Patients also commented during interviews that they rarely had the chance to speak with health professionals because they were busy doing other activities and were often away from the bedside. Before patient transfer between sites, nurses prioritised organising transportation and providing clinical handover by telephone to other health professionals at the receiving health care setting, completing discharge documentations, or taking care of other patients' immediate needs. Nurses were observed to undertake multitasking activities that prevented them from advocating for older patients' preferences about any medication changes made. In these multitasking activities, the researchers observed nurses undertake indirect patient care, which included preparation of paperwork for patient discharge, and making telephone calls to facilitate the arrival or departure of various patients. These activities indicated that the discourse of organisational efficiency took precedence over the discourse of eliciting preferences during older patients' transfers. These activities indicated that the discourse of organisational efficiency took precedence over the discourse of eliciting preferences during older patients' transfers. Further, the structure of the interdisciplinary ward rounds hindered eliciting older patients' preferences about medications. In the study hospitals, interdisciplinary ward rounds including medical, nursing, allied health professionals and pharmacists, were conducted each morning and some afternoons. When the team visited patients at the bedside during ward rounds, the primary focus was on discussing with patients about their clinical issues instead of eliciting patients' preferences about their medications. During bedside visits, the medical consultant led the communication by asking other health professionals' opinions when their contributions were needed. After completion of the bedside ward rounds, the interdisciplinary team tended to gather around the ward reception area or satellite nurses' station to review each patient's clinical situation and make changes to their medications, which left no opportunity for older patients to be involved in these decision‐making processes.

#### 3.1.2 Older patients' beliefs about effectiveness of medications

Older patients had varying beliefs about taking medications after being transferred between settings. These varying beliefs were that certain medications were ineffective or unnecessary, that specific forms of medications such as injections may cause more harm than good.

During an interview, a 70‐year‐old patient admitted to the geriatric rehabilitation setting expressed the view that doctors provided insufficient information about reasons for continuing her Parkinson's medication. She believed that taking Kinson^®^ (levodopa and carbidopa) did not effectively manage her reduced ability to move caused by progressive Parkinson's disease, and therefore perceived she no longer needed to keep taking the medication. Nonetheless, the patient agreed to take her medication regardless of her beliefs about its lack of effectiveness; an acknowledgement of the doctor's authority in organising the prescription:

I just don't think they're terribly effective, that's all. And I would wonder why he said to me to continue taking them if it's not ‐ I've read, with the description of the disease, that it's not terribly effective. So why am I still taking it? And what's it doing for me? I don't know. That's the neurologist though. (Pt28_Geri_Rehab1)

Some older patients wanted to avoid taking specific dosage forms of medications because they believed that their risks outweighed potential benefits. These patients received insufficient explanation or varied advice from different health professionals about these medications when moving across transitions. One 82‐year‐old patient who transferred from the acute setting to the geriatric rehabilitation setting reflected on the conflict she had with health professionals about taking vitamin B12 injections:

Because I was given penicillin and I was allergic to it and I was given the derivative, which is called Flopen (flucloxacillin), and there are other derivatives, I've looked in the doctors' books. And I had very bad consequences and I [therefore] don't want vitamin B12 pumped into me at a massive rate, and they won't tell me, is it injections, is it liquid or is it tablets, what is it? (Pt33_Subacute1)

The patient's resistance to taking the vitamin B12 injection was associated with her belief that an injection could cause an allergic reaction. Since the patient had previously experienced an allergic reaction from a penicillin injection, she was reluctant to have further injections. Nursing and medical staff reportedly did not perceive that conveying information to the patient about the vitamin B12 injection was important.

### 3.2 Establishing patient‐centeredness in medication communication

The strategies that health professionals used to establish patient‐centeredness in medication communication comprised empathetic talk to prioritise patient preferences, informative talk to clarify patients' medications concerns, and encouraging talk to enhance shared decision‐making.

#### 3.2.1 Empathetic talk prioritising patient preferences and needs

In this research, empathetic talk was defined as a communication strategy used by health professionals to connect with older patients by acknowledging their emotional state, such as anxiety, stress, or health care concern, as a result of medication‐related issues. For older patients prescribed multiple medications and who experienced several changes to their regular medications during transitions of care, empathetic talk occurred when health professionals displayed sensitivity about patients' preferences and concerns through acknowledging and reiterating them by, for example, repeating back to older patients what they have expressed about those changes. Health professionals used empathetic talk with a diverse range of patients whose medication preferences needed to be unveiled, including those with speech problems and from patients who were not fluent in English by providing them with enough time to express themselves during conversations, which gave those patients a sense that their concerns and opinions mattered. Pharmacists were observed to engage in empathetic talk with older patients, especially upon admission or during discharge medication education. However, nurses and doctors were generally not observed to use empathetic talk in their interactions with patients. Observations revealed that there were cases where older patients reported that they were unhappy with the new medication regimens prescribed by doctors after admitting to the hospital, which prompted pharmacists to engage the patient with empathetic talk when communicating to the patients the rationale behind the prescribed changes.

The following observation excerpt demonstrated the discharge communication between the pharmacists and 81‐year‐old patient. The patient did not want to stay an additional day at hospital, which made him very upset, therefore challenged the medical team's decision about the discharge date. He expressed his anxiety about his new insulin regimen since he believed that the previous doses were higher and worked better than the ones prescribed at the hospital by the endocrinologist. Later on the same day, the endocrinologist team made a quick decision about the patient's insulin regimen and increased both his morning and night doses. Although the patient's interest was being served in this situation as he convinced the medical team to discharge him earlier and to change his insulin doses, this increased the pharmacist's concern about insulin management after discharge since he lived alone. During the pharmacist's last visit, there was discussion with the patient about final insulin changes made to his discharge medications:

*Pharmacist educates the patient on the new insulin regimen in the beginning of the conversation*.

**Pharmacist:** So you might be happy about this, we've increased your insulin doses.

**Patient:** To?

**Pharmacist:** Well, 24 in the morning.

**Patient:** Perfect, OK.

**Pharmacist:** And 20 at night time.

**Patient:** Ok.

**Pharmacist:** I thought the insulin would be the one that you were most anxious about, so I thought we'd talk about that first. And you are on the safety net [above the safety net, patients receive medications at a markedly reduced cost or free of charge]. So you've got a full supply of insulin to keep in your fridge at home.

**Patient:** Thank you. Thank you.

**Pharmacist:** It would be just for the next couple of days because we've changed the dose, just make sure you double check. Because I know, it might be easy if you go home and you just get into the habit of dialling up your old dose.

**Patient:** Okay.

**Pharmacist:** And we'll show‐ when your daughter comes to visit (*to patient*'*s home*), show her the list and show her the medications as well. Just good for someone else to know what is going on, I think. Because, if you ever have to come to the hospital or something, then you have got some family member that can tell us what you take.

**Patient:** Excellent. Thank you very much.

*Excerpt Code: Pharm5_Obs_Subacute2_Interaction15_Pt110

The insulin dosages that the medical team prescribed were slightly lower than those the patient had previously taken at home. The pharmacist engaged in empathetic talk by explicitly acknowledging the patient's feelings about the changes to his insulin regimen (*I thought the insulin would be the one* ***that you were most anxious about***). Therefore, she prioritised his needs of being educated on the new insulin regimen. To alleviate any possible financial distress, the pharmacist reassured him that the insulin supply was free of charge. The pharmacist practised the discourse of safety throughout the interaction and demonstrated cognitive empathy by helping the patient to alleviate his anxiety by imagine how his insulin management could occur following discharge (***it might be easy*** *if you go home and you just* ***get into the habit of*** *dialling up your old dose*). The pharmacist was very concerned about the patient's potential insulin overdose following discharge, therefore she advised the patient to inform this daughter about the discharge medications.

#### 3.2.2 Informative talk provided by health professionals

Informative talk occurred when health professionals provided older patients with tailored education about medications in a way that they could understand. Older patients reported needing regular discussions with health professionals about medications, particularly when they were given a new medication after hospital transfer. Some patients felt dissatisfied with the amount of information given and they reported that they did not receive the information they required. These patients involved those who managed medications independently at home and those who felt responsible in keeping track of the medication changes that occurred across transitions. These patients prompted health professionals to provide more information about medications. Some who were admitted to the acute settings with complex health conditions and complained about memory issues indicated that they preferred not to have an informative talk with health professionals at hospital admission since they were too sick to retain information. However, they wanted information once their health started to improve, and they became more capable of discussing their point of view.

The following excerpt demonstrated informative talk between a non‐English speaking 91‐year‐old patient and a registrar (a doctor undergoing training to become a specialist) regarding the importance of continuation of medication treatments for her lymphedema:

**Patient (interpreter):** (Patient speaking) I was really struggling to walk around (Patient speaks) Every hour or so I need to go to the toilet (Patient speaking) due to the medications that I'm taking.

**Doctor:** You're still not on very many (*refers to medications*)!

**Patient (interpreter):** But it is more now than it was.

**Doctor approves:** Yeah, a little bit more.

**Doctor: But, so‐** *(Patient speaks)*

**Patient (interpreter):** So I'm really noticing a lot of pain up here.

**Doctor:** So…so all this excess fluid, hmmm…and the shortness of breath we think is being caused by the one process. Which is the heart not pumping blood around.

**Interpreter:** I'm just explaining that my heart is beating a lot. *(Patient speaks)*.

**Doctor**: You still have a lot of excess fluid in your body that we need to…to get rid of, which is why you are going to the toilet so much, which is a good thing. (*Interpreter translates to the patient)*.

**Doctor continues:** That will slow down over time once the fluid comes off.*‐Interpreter translates to patient‐* We only continue whatever medications are necessary ahm…I think they're making a difference. (*Patient speaks*)

**Patient (interpreter):** So, you mean the fluids are coming out by me going to the toilet, so I need to [keep going to the toilet]?

**Doctor:** No, no, no, no! I am saying that the way that your body gets rid of the excess fluid is by urinating. **(** *Interpreter translates to patient)*

**Doctor**: So, you going off to the toilet very frequently is a sign that the medication is doing its job. **(** *Interpreter translates to patient)*

*Excerpt code: Med3_Obs_MedWard2_30818_Pt36

Local policy of the hospitals indicated that interpreting services could be undertaken either by telephone or face‐to‐face. After health professionals established the need for an interpreter during patients' admissions, these health professionals should be able to make bookings through the hospital system as needed. However, organising the interpreter service to inform the patients from non‐English speaking backgrounds about medications was rarely observed during ward rounds. This lack of interpreter availability made older patients reliant on family members or other patients in the same room who spoke the same language to communicate to health professionals. On this occasion, an individualised approach using the interpreter facilitated medication information exchange since the family member was not present during the ward round. In this observation, a registrar and a consultant visited the patient after the interpreter arrived at the ward, but only the registrar was involved in discussion whereas the consultant sat away from the bedside. The older patient believed that her frequent urination was a repercussion of taking several medications at the hospital. The registrar used words that weakened the force of her statements (*a little bit more*), which may have mitigated the patient's emotional distress caused by medications administered at hospital. The registrar acknowledged the patient's concerns and provided a positive evaluation of what the patient perceived as an unpleasant experience by saying, ‘you are going to the toilet so much, which is a good thing’. While the registrar engaged in informative talk throughout the conversation, she avoided medical language and used everyday social language to enable patient's understanding of the link between her frequent urination and taking furosemide (diuretic). Further, the registrar never mentioned the generic or medication brand name to the patient. At the end of conversation, the registrar provided an explanation such as ‘you going off to the toilet very frequently is a sign that the medication is doing its job’. This information was strategic in calling the patient's attention to positive effects of taking the medication (furosemide) instead of negative experiences caused by taking it.

Observations revealed that nurses' use of informative talk rarely happened during medication administration. Nurses prioritised their efforts in preparing and administering medications rather than providing informative talk during these medication activities. Our observations explored that nurses tended to engage with informative talk when they administered particular medications including laxatives, painkillers or inhalers. The following interaction was one of the rare examples where the nurse did engage in informative talk with a 68‐year‐old patient during medication administration:

**Registered Nurse:** And the doctor also charted you Coloxyl^®^. It's a laxative, it just helps to open your bowels. Because you are taking so many pain medication, analgesic, it will make you constipated. Alright, and we don't want that complication, so it's very…actually really, really important. (Patient 12: Ok) So you happy if I just add two of those little brown tablets? They are quite gentle. We'll start off with that. Because we just really want you to have a regular, daily bowel movement. Because you don't want another complication. Because if that's really compacting the tummy it can make your pain worse. Now and I'll also have a look, is there any more pain medication I can give you.

**Patient:** And the lady (refers to the pharmacist) that came in saying, she is the pharmacist, she was talking about some lozenges for nicotine treatment.

**Registered Nurse:** Ok, yeah. (Pause) Do you want a break from the oxygen? The nasal prongs?

*Excerpt Code: RN1_Obs_WardMed1_Interaction10_Pt12

The nurse talked on behalf of the doctors to provide the rationale behind why they prescribed Coloxyl^®^ and Senna (docusate sodium ‐ laxative) upon the patient's admission to hospital and emphasised its importance as part of pain management. While informing the patient, the nurse frequently used the plural personal pronoun (i.e. ‘we’), which indicated adoption of a partnership discourse to convince the patient to take the tablets. However, the nurse dominated the talk by having interactional control over the medication conversation and no effort was made to include the patient. In this interaction, there were conflicting medication interests between the patient and the nurse since the nurse was trying to provide information about laxatives but the patient was interested in obtaining further information about the lozenges for nicotine treatment.

#### 3.2.3 Encouraging talk to enhance shared decision‐making

Encouraging talk referred to using inclusive communication strategies with a focus on motivating older patients to be involved in medication decisions. Health professionals engaged in encouraging talk particularly when they wanted older patients to take responsibility in managing their own medications, especially following discharge. Health professionals' encouraging talk occurred with older patients whose medications needed to be altered after discharge, with patients whom pharmacists believed would require dose administration aids to support the taking of their medications in the future, or with patients who were hesitant in taking particular medications. Observations revealed that encouraging talk rarely happened with older patients who were slightly confused, or not fluent in English or who had hearing impairments. With these patients, health professionals attempted to involve family members if they were present at the bedside.

Doctors encouraged older patients to be involved in decisions about medications by seeking their preferences in situations where doctors made changes to patients' regular medications or when they decided to prescribe a new medication for acute symptoms such as pain or constipation:

**Registrar**: We can start some Fibogel^®^ or Metamucil^®^. (to patient) Have you ever used Metamucil^®^?

**Patient**: Yeah, I've got it at home.

**Registrar**: Do you find it works?

**Patient**: I can't recall.

**Registrar**: Will we try it?

**Patient**: (responds affirmatively)

**Registrar**: You've got to have a lot of water with it.

**Patient:** Is it orange?

**Registrar**:…might be a different brand but same sort of thing…

*Excerpt code: Med6_Obs_ Geri_Rehab4_Interaction_7

In this excerpt, the patient was a 78‐year‐old man with constipation. Doctors consulted with the patient about the plan for laxatives. The registrar used the questioning strategies to encourage the patient to provide more information about his experience of using laxatives at home. Although the patient did not recall his previous experience with Metamucil^®^, the registrar used a partnership discourse (*Will we try it?*) to be more inclusive for the patient to participate in decision‐making. Although the registrar engaged in encouraging talk, it was not clear that this attempt led to shared decision‐making between the doctor and the patient, because the patient's contributions to the conversation remained very minimal. The doctor's use of closed‐ended questions only required the patient to approve or disapprove his suggestions rather than facilitating further input from the patient.

Nurses engaged with encouraging talk when older patients sought to avoid taking particular medications or when nurses wanted older patients to take responsibility to express their medication concerns or when nurses asked older patients to direct medication requests to doctors or pharmacists during their bedside visits. Pharmacists tended to use encouraging talk with older patients during discharge education to prompt them to discuss continuation or alterations of specific medications prescribed in hospital with their general practitioners. Encouraging talk was demonstrated by the pharmacist who visited an 80‐year‐old patient directly after his admission to the geriatric rehabilitation facility. The patient was diagnosed with hand gout, which contributed to difficulties in managing his asthma inhaler:

**Pharmacist:** Ok. So…other than your packed medications, you will have a couple of things that is not in a Webster pack.

**Patient:** Yeah, [inaudible]

**Pharmacist:** Yeah so you've got your inhaler…

**Patient:** Yeah, that's what I am trying to say.

**Pharmacist**: (laughs) And some part of using one of those inhalers will require some strength in your hands.

**Patient:** Yes, I found it troubling with the Seretide^®^ (fluticasone and salmeterol), because this hand doesn't work as well. But I managed by putting them both together. But still uncomfortable.

**Pharmacist:** Do you know what I think we'll do while you're here, is get a spacer and show you how to use one of those. Because that gives you a little bit more time.

**Patient:** Yeah, yeah but I still have to do the squeezies (He refers to Seretide^®^).

**Pharmacist:** Yes. I think overall you could find more something else to push it. We'll give it a go. We'll have a look.

**Patient:** We'll give it a go, we'll give it a go.

**Excerpt code:** Pharm5_Obs_ Geri_Rehab2_Interaction1_Pt53

The patient expressed his concerns about being unable to use his inhaler effectively due to weakness of his handgrip. The pharmacist's statement, (*Do you know what I think we'll do while you're here*…), was a strategy to encourage the patient to think about learning how to use a spacer as an alternative option to the use of inhaler. The patient responded by using first‐person plural pronoun and mirroring what the pharmacist had said (*we'll give it a go*), which was a sign of agreement between the patient and the pharmacist. The pharmacist had this interaction at the bedside on the same day that the patient was admitted to the hospital and her talk was encouraging in a way that the patient felt motivated to practise using the spacer during his hospital stay.

## 4 DISCUSSION

Patient‐centred communication about medications was impacted by both older patients and health professionals. Patient preferences, experiences and beliefs about medications and health professionals' use of communication strategies such as empathetic, informative and encouraging affected the provision of patient‐centred communication across transitions of care. Health professionals used these various communication strategies to understand patients' individual preferences, to tailor the medication information to patients' needs and knowledge levels, and to encourage their involvement in decision‐making. Older patients demonstrated changing preferences for involvement in medication decisions and having control of managing their own medications across transitions.

There were challenges for health professionals in establishing patient‐centeredness in medication communication across transitions of care. These challenges involved the absence of family members of patients who were slightly confused, with complex health conditions or patients who were not fluent in English, lack of access to interpreter services. Past work emphasised that greater attention is needed by health professionals to involve family members in medication communication because they demonstrated active and shared decision‐making as to how their older relatives' medications were managed at the point of care transitions. Family members also played the role of interpreter for those who were not fluent in English (Manias et al., 2019). Competing interests between patients and health professionals, health professionals' multitasking activities or older patients' preference to take a passive approach in medication decisions at transition points of care constituted additional challenges to patient‐centred medication communication. Similarly, previous research showed that a task‐centred approach based around the routines created psychosocial distance between nurses and patients by giving an impression of being ‘too busy’, which was conflicting with a patient‐centeredness in nursing practice (O'Hagan et al., 2014). As recently suggested by Tobiano et al. (2021), encouraging older patients to articulate their medication concerns and preferences could foster shared decision‐making across transitions; however, a closed style of questioning by health professionals was common.

Our findings suggest that it was important for health professionals to elicit older patients' beliefs about medications soon after their admission to hospital. Previous research showing that older patients' compliance with medication regimens after discharge was often influenced by their beliefs regarding their perceived necessity (Allen LaPointe et al., 2011), or benefits or side effects of prescribed medications (Souter et al., 2014). Our study showed that, in the situations where older patients were not informed about the rationale for new prescribed medications before discharge, patients became sceptical about needing these medications. Most older patients seemed hesitant to verbalise their opinions concerning prescribed or altered medications since they did not want to conflict with medical authority in decision‐making. Similarly, older patients' hesitancy to speak up about their medications was reported by Bagge et al. (2014), which emphasised that older patients believed the hospital is not the place to question medical staff about their decisions. Insufficient communication between older patients and health professionals about the necessity and benefits of the prescribed medications contributed to a lack of concordance of opinions between older patients and health professionals, which interfered with enabling patient‐centred communication.

Patient preferences fell along a spectrum ranging from patients as active participants preferring to involve themselves in decisions, to passive participants, deferring responsibility for making decisions to health professionals. This finding supports previous investigations that showed older patients did not wish to be involved in decision‐making processes in hospitals, whereas some patients demonstrated involvement ranging from sharing information to active participation in decisions (Belcher et al., 2006; Bucknall et al., 2019). We found the way that older patients positioned themselves as an active or passive participant changed as they moved across care transitions. These differences can be attributed to older patients' perception of health professionals' availability in the inpatient environment. Similarly, previous literature also showed that health professionals' attitudes during patient encounters showed that they maintained control in sharing medication knowledge and did not empower patients to ask questions, which contributed to patients becoming passive recipients. Our findings similarly demonstrated that receiving insufficient information or contradictory medication advice from multiple health professionals during hospital stay seemed to hamper older patients' active involvement, which, in turn, contributed to paternalistic practice where health professionals played dominant roles in decision‐making processes. For example, past research demonstrated that patients' previous disempowering experiences such as lack of time and information given to the patients by doctors and nurses in relation to their medications discouraged patients to be active recipients of care at the hospital (Doherty & Doherty, 2005; Ringdal et al., 2017). As indicated in past studies, our findings demonstrated that patients' preferences for passive involvement are associated with their perception of power disparity between themselves and health professionals, and inadequacies of health professionals' interpersonal communication practices such as inattentive listening or showing disinterest (Halvorsen et al., 2020; Ringdal et al., 2017).

Our observations also revealed missed opportunities for health professionals to establish patient‐centeredness in medication communication during care transitions. Pharmacists usually encouraged older patients to talk about their preferences, and pharmacists combined empathetic talk within a discourse of partnership that gave patients the impression that their opinions regarding medication decisions mattered. However, there were times when empathetic talk was not enough to fully involve older patients in medication communication. This situation was notable when patients felt rushed at the time of discharge, and medication communication tended to occur in more one‐sided and haphazard ways where pharmacists gave a large amount of information without ensuring patients' understanding and preferences. Furthermore, engaging in empathetic or encouraging talk with older patients about their medications appeared challenging for nurses and doctors. Many nurses were observed to prioritise the discourse of task completion over the discourse of eliciting preferences before discharge, which prevented them from taking a patient‐centred approach. Nurses also perceived that communication about medication with patients after admission or before discharge was the pharmacists' role rather than their role (Bolster & Manias, 2010). With respect to communication with doctors, this tended to occur during ward rounds, where doctors could allocate shorter periods of time for each patient compared to other health professionals, which created limited opportunity for them to engage in empathetic or informative talk with patients regarding their medications. Although doctors were engaged in informative talk about patients' treatments during bedside visits, missed opportunities were likely to occur when they did not adequately elicit older patients' views about discharge medications (Allen et al., 2018).

### 4.1 Limitations

A limitation of this study is the potential for the observer effect to occur in participating health professionals during their communication with patients. However, researchers spent extensive periods with participants, which helped to gain rapport and familiarity. Researchers also emphasised their intention to understand complexities with medication communication in older patients rather than judging practice.

## 5 CONCLUSION

Our findings elaborated patient preferences, experiences and medication beliefs as characteristics that need to be considered by health professionals when providing individualised medication communication to older patients in the context of care transitions. Shared decision‐making was more likely to happen when health professionals adopted encouraging talk where they used a partnership discourse and inclusive statements during medication communication. Engagement with empathetic talk during transitions of care was helpful for health professionals to reduce unequal power relations between themselves and older patients, which, in turn, enabled older patients to communicate more confidently about their medication preferences.

## 6 RELEVANCE TO CLINICAL PRACTICE

While communication skills are taught in university programmes, health professional students are often not taught about the communication skills relating to helping patients and families to manage medications in clinical practice across transitions of care, as demonstrated by the missed opportunities identified in the study (Manias & Bullock, 2002). Health professionals should identify older patients' preferences for both managing medications and including themselves in medication communications at different time points during their movement from admission to discharge. In some cases where the patient turnover is high such as in acute care settings, health professionals need to be aware of that they do not prioritise the discourse of organisational efficiency over the discourse of eliciting older patients' medication preferences. Health professionals need to organise education sessions with family members or interpreters for older patients with language barriers or cognitive issues. Organisational commitment is required to explore older patients' beliefs regarding medications because their beliefs can determine whether patients would take and manage their medications correctly during the post‐discharge period. It was observed that the healthcare environment involves many organisational tensions in communication across transitions of care. Therefore, communication about medications should be accepted as everyone's responsibility regardless of health professionals' roles and it should be undertaken regularly during patients' hospitalisation. Health professionals can benefit from various individualised communication strategies to ensure that patients' beliefs and views are understood, their medications are adjusted as necessary and eventually there is a shared medication understanding between older patients and health professionals.

## CONFLICT OF INTEREST

The authors declare that there is no conflict of interest.

## AUTHOR CONTRIBUTIONS

GO and EM made substantial contribution to designing and drafting of the manuscript. GO, EM and were responsible for data collection/analysis and data interpretation. EM, TB, CJ, CH, RW and made critical revisions to the paper for important intellectual content. EM, TB, CJ, CH and RW obtained funding. All authors have agreed on the final version of the review.

## Supporting information

Appendix S1

## Data Availability

The data that support the findings of this study are available on request from the corresponding author. The data are not publicly available due to privacy or ethical restrictions.
