# Supplementary material for: Towards patient‐centred communication in the management of older patients' medications across transitions of care: A focused ethnographic study
Source: J Clin Nurs. 2021 Dec 6;31(21-22):3235–49. doi: 10.1111/jocn.16162 (PMC9786755; doi:10.1111/jocn.16162)
Supplement: Supplementary file 1 — Appendix S1 [file JOCN-31-3235-s001.docx]

**Appendix S1: Consolidated criteria for reporting qualitative studies (COREQ): 32-item checklist**

Developed from: Tong A, Sainsbury P, Craig J. Consolidated criteria for reporting qualitative research (COREQ): a 32-item checklist for interviews and focus groups. *International Journal for Quality in Health Care*. 2007. Volume 19, Number 6: pp. 349 – 357

**YOU MUST PROVIDE A RESPONSE FOR ALL ITEMS. ENTER N/A IF NOT APPLICABLE**

| **No. Item** | **Guide questions/description** | **Reported on Page #** |
| --- | --- | --- |
| **Domain 1: Research team and reﬂexivity** |  |  |
| *Personal Characteristics* |  |  |
| 1. Interviewer/facilitator | Which author/s conducted the interview or focus group? | 6 |
| 2. Credentials | What were the researcher’s credentials? E.g. PhD, MD | It has included in the title page, PhD-student, Dr, Adjunct Professor, Professor, Research professor |
| 3. Occupation | What was their occupation at the time of the study? | 6 |
| 4. Gender | Was the researchers male or female? | 7 |
| 5. Experience and training | What experience or training did the researcher have? | 7 |
| *Relationship with participants* |  |  |
| 6. Relationship established | Was a relationship established prior to study commencement? | 7 |
| 7. Participant knowledge of the interviewer | What did the participants know about the researcher? e.g. personal goals, reasons for doing the research | 7 🡪 We explained that researchers did not have a prior relationship with any of participants in this study. |
| 8. Interviewer characteristics | What characteristics were reported about the interviewer/facilitator? e.g. Bias, assumptions, reasons and interests in the research topic | 7 |
| **Domain 2: study design** |  |  |
| *Theoretical framework* |  |  |
| 9. Methodological orientation and Theory | What methodological orientation was stated to underpin the study? e.g. grounded theory, discourse analysis, ethnography, phenomenology, content analysis | 4🡪 We have provided details about the focused ethnography under research design. |
| *Participant selection* |  |  |
| 10. Sampling | How were participants selected? e.g. purposive, convenience, consecutive, snowball | 5 |
| 11. Method of approach | How were participants approached? e.g. face-to-face, telephone, mail, email | 5 |
| 12. Sample size | How many participants were in the study? | 8 |
| 13. Non-participation | How many people refused to participate or dropped out? Reasons? | 6🡪 There was not any patient who dropped out the study, we haven’t counted the number of patient who refused to participate into the study. |
| *Setting* |  |  |
| 14. Setting of data collection | Where was the data collected? e.g. home, clinic, workplace | 5 |
| 15. Presence of non-participants | Was anyone else present besides the participants and researchers? | No |
| 16. Description of sample | What are the important characteristics of the sample? e.g. demographic data, date | 5 |
| *Data collection* |  |  |
| 17. Interview guide | Were questions, prompts, guides provided by the authors? Was it pilot tested? | 29 🡪 Yes, we had interview, observations and focus group schedules that we have used in this study. These schedules were summarized in the Table 1. |
| 18. Repeat interviews | Were repeat interviews carried out? If yes, how many? | No |
| 19. Audio/visual recording | Did the research use audio or visual recording to collect the data? | 6 |
| 20. Field notes | Were ﬁeld notes made during and/or after the inter view or focus group? | 6 |
| 21. Duration | What was the duration of the interviews or focus group? | 6 |
| 22. Data saturation | Was data saturation discussed? | 6-7 |
| 23. Transcripts returned | Were transcripts returned to participants for comment and/or correction? | N/A |
| **Domain 3: analysis and ﬁndings** |  |  |
| *Data analysis* |  |  |
| 24. Number of data coders | How many data coders coded the data? | 7🡪 Two |
| 25. Description of the coding tree | Did authors provide a description of the coding tree? | Demonstrated as codebook, including data analysis questions in Table 2. Page 31-32. |
| 26. Derivation of themes | Were themes identiﬁed in advance or derived from the data? | 7-8 |
| 27. Software | What software, if applicable, was used to manage the data? | 7, NVivo 12 (QSR Melbourne) |
| 28. Participant checking | Did participants provide feedback on the ﬁndings? | No |
| *Reporting* |  |  |
| 29. Quotations presented | Were participant quotations presented to illustrate the themes/ﬁndings? Was each quotation identiﬁed? e.g. participant number | Yes, 9-21 |
| 30. Data and ﬁndings consistent | Was there consistency between the data presented and the ﬁndings? | Yes, 9-21 |
| 31. Clarity of major themes | Were major themes clearly presented in the ﬁndings? | Major discursive practices were clearly presented in Figure 1. |
| 32. Clarity of minor themes | Is there a description of diverse cases or discussion of minor themes? | 9-21, All the positive and negative experiences of participants were explained throughout findings to demonstrate the variations. |

**Once you have completed this checklist, please save a copy and upload it as part of your submission. When requested to do so as part of the upload process, please select the file type: *Checklist*. You will NOT be able to proceed with submission unless the checklist has been uploaded. Please DO NOT** **include this checklist as part of the main manuscript document. It must be uploaded as a separate file.**
